# Long‐term development of species richness in a central European beech (*Fagus sylvatica*) forest affected by windthrow—Support for the intermediate disturbance hypothesis?

**DOI:** 10.1002/ece3.8028

**Published:** 2021-08-16

**Authors:** Peter Meyer, Marcus Schmidt, Eike Feldmann, Jürgen Willig, Robert Larkin

**Affiliations:** ^1^ Department Forest Nature Conservation Northwest German Forest Research Institute Hanoversch‐Münden Germany; ^2^ Department Forest Development and Environment State Forest Enterprise HessenForst Gießen Germany; ^3^ Department Growth and Yield Northwest German Forest Research Institute Göttingen Germany

**Keywords:** biodiversity, competition, disturbance, forest management, habitat heterogeneity, succession

## Abstract

On the basis of long‐term surveys of permanent plots and traps, we examined the communities of saproxylic beetles, fungi, herbs, and trees on an untreated 22 ha large beech forest windthrow and asked whether the results lend support to the intermediate disturbance hypothesis (IDH). We studied species richness and the similarity of community composition. Additionally, we grouped species by their frequency trend over time to successional model types to examine whether, corresponding to the IDH, the diversity of these groups explained peak richness at intermediate intervals after the disturbance. In line with the IDH, species richness showed a hump‐backed temporal course for alpha and gamma diversity. We found evidence for a linear succession directly after the disturbance. This, however, did not continue, and in all species groups, a partial recovery of the initial community was observed. In the case of fungi, herbs, and trees, but not for saproxylic beetles, alpha diversity was driven by the diversity of the successional model types. Our results underline that the mechanisms driving species richness after disturbances are more complex than the IDH suggests and that these mechanisms vary with species group. We assumed that, besides competition, legacy effects, facilitation, habitat heterogeneity, and random saturation of the species pool are important. In case of trees and herbs, we found indications for strong legacy and competition effects. For fungi and beetles, substrate heterogeneity and microclimate were assumed to be important. We concluded that disturbances contribute to increasing species richness not only by reducing the effectiveness of competitors but also by increasing the amount and diversity of resources, as well as their rate of change over time.

## Graphical abstract

On the basis of long‐term surveys, we examined the communities of xylobiotic beetles (a), fungi (b), herbs (c), and trees (d) on an untreated beech forest windthrow and asked whether the results lend support to the intermediate disturbance hypothesis (IDH). The results underline that the mechanisms driving species richness after disturbances are more complex than the IDH suggests and that these mechanisms vary with species group. We concluded that disturbances contribute to increasing species richness not only by reducing the effectiveness of competitors but also by increasing the amount and diversity of resources, as well as their rate of change over time.

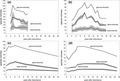

## 1 INTRODUCTION

A disturbance can be defined as “… any relatively discrete event in time that disrupts ecosystem, community, or population structure and changes resources, substrate availability, or the physical environment.” (Pickett & White, 1985). Disturbances, such as windthrow, insect infestation, flooding, or fire, are a significant element of the natural forest dynamic (Čada et al., 2016; Nagel & Diaci, 2006; Nagel et al., 2007), and forestry activities are deemed to be close‐to‐nature if they are analogous to natural disturbances (Long, 2009; Seymour et al., 2002). With ongoing climate change, disturbance patterns may change, with an increase in disturbance frequency and/or intensity likely in some areas (Seidl et al., 2017). As the type, extent and frequency of environmental disturbances have a considerable impact on stand structural characteristics (White et al., 2015) and biodiversity (Connell, 1978), understanding the link between disturbance and diversity has great relevance for forest management (Gosper et al., 2013; Meyer & Ammer, 2019; Wohlgemuth et al., 2002).

Among many different models to explain diversity–disturbance relationship (Wilson, 1990), the intermediate disturbance hypothesis (Connell, 1978, s. also Horn, 1975) is the most influential. According to the intermediate disturbance hypothesis (IDH), biodiversity peaks at an intermediate time span after the disturbance (also at intermediate levels of disturbance frequency or intensity). The basic reasoning behind the IDH is that disturbances set back competitive exclusion and open up space for new species to colonize and exploit resources (Connell, 1978). Colonizers coexist for some time with survivors and competitive species, and this coexistence results in an increase in diversity up to intermediate periods of time. As time passes, competitive exclusion leads to species loss, and thus, richness decreases again. These processes produce a distinct peaked (hump‐backed) curve of species diversity after the disturbance event. Since the first empirical reproduction of the IDH (Sousa, 1979), the hump‐backed curve of species diversity after a disturbance has been found in many studies (Mandryk & Wein, 2006; Molino & Sabatier, 2001; Peterson & Reich, 2008; Peter & Harrington, 2009; Yuan et al., 2016).

Although the IDH was at first widely accepted, it has recently been the subject of renewed debate (Fox, 2013a, 2013b; Sheil & Burslemm, 2013), with Fox (2013a) calling for the hypothesis to be abandoned due to logical inconsistencies in the mechanisms proposed as drivers of increase and decline in diversity. In literature meta‐analyses, both Mackey and Curie (2001) and Svensson et al. (2012) found as few as 16% of studies supported the IDH hypothesis as an explanation for observed diversity. Another review, however, which concentrated on studies in terrestrial systems (Kershaw & Mallik, 2013) reported 46% support for the IDH, with several studies in forest ecosystems among those for which the IDH fitted observed diversity patterns. Also, the likelihood of a peaked diversity–disturbance curve seems to be greatest if species richness and time since disturbance are the factors considered (Mackey & Curie, 2001). Connell (1978) concluded that different diversity–disturbance relationships are not mutually exclusive and several may simultaneously contribute to diversity, with IDH being by far the most important.

In many biomes, positive biodiversity effects of unlogged disturbed forest areas have been shown (Lindenmayer et al., 2008; Lindenmayer & Noss, 2006; Thorn et al., 2018). High levels of species richness are often typical for early successional stages taking place after disturbances in forests (Hilmers et al., 2018), although their significance for biodiversity conservation is still widely underrated (Halpern, 1989; Swanson et al., 2011).

Wind is the most important natural disturbance agent in Central Europe (Čada et al., 2016; Gardiner et al. 2010), and to date the most researched. In particular, the storms “Vivian and Wiebke” in February 1990 gave rise to intense research effort on windthrow in Switzerland and Germany (Fischer & Fischer, 2009; Nationalparkverwaltung Bayerischer Wald, 2001; Schönenberger et al., 1995; Wohlgemuth & Kramer, 2015). In parallel with the findings in other biomes, the studies showed positive effects of windthrow and bark beetle attacks on biodiversity, for example, in the Bavarian National Park (Bässler & Müller, 2010; Bässler et al., 2012; Lehnert et al., 2013). While these studies were concentrated on Spruce and montane mixed forests, a study of beech forests with windthrow echoed these findings (Lachat et al., 2016).

Although broadleaved forests constitute the major part of natural vegetation in Central Europe (Bohn & Neuhäusl, 2000), with beech forests as the most important type, investigations on the biodiversity effect of intermediate‐severity disturbances in these forests are rare (exceptions: Kompa & Schmidt, 2003, 2005; Willig, 2002), and long‐term effects have barely been studied. This knowledge gap is of particular relevance as even in European beech (*Fagus sylvatica*) forests, intermediate‐severity disturbances are more common than previously thought (Nagel et al., 2007). This was illustrated recently with beech dieback also observed in larger areas as a consequence of the drought years 2018 and 2019 (Schuldt et al., 2020).

In Germany, in South East Hesse, the storms Vivian and Wiebke also caused extensive damage in broadleaved forests. With a windthrow area of 22 ha, the “Weiherskopf” was by far the worst affected of all the strict forest reserves in Hesse (Willig, 2002). Tree roots were not held fast enough in the saturated but unfrozen forest soil to resist the loosening effect of the repeated storms, resulting in extensive windthrow areas (Winterhoff et al., 1995). This windthrow event at “Weiherskopf” fits the definition of a medium severity disturbance event (Connell, 1978; Hart & Kleinman, 2018).

Our study aims to examine the long‐term effect of an untreated windthrow area on species richness in “Weiherskopf” in the time since the storms Vivian and Wiebke. We had the unique opportunity to draw on a substantial database of permanent plot surveys of saproxylic beetles and fungi, herbs, and tress. Monitoring data from a first research phase, spanning from 1990 to 2000 (mycological study until 2008), were extended by own surveys for herbs and trees in 2013/14. Time series were constructed for woody plants and herbs (both 23 years), wood‐dwelling fungi (17 years), and saproxylic beetles (9 years).

To evaluate whether and, if so, to what extent untreated disturbance areas in broadleaved forests contribute to conserving biodiversity, it is important to rely on a sound theoretical foundation. Therefore, we tested whether the course of species richness of beetles, fungi, herbs, and trees at the windthrow area in the strict forest reserve “Weiherskopf” corresponds to the IDH. In particular, we hypothesized that after the disturbance:

1. The development over time of alpha, beta, and gamma species richness would follow a hump‐backed curve characteristic of the IDH.
2. The similarity of species composition would decrease monotonically in the course of time indicating a succession. The change of species composition results in distinct successive phases.
3. Species can be classified by their frequency trend over time. There are groups of species showing a distinct decreasing, increasing or hump‐backed (optimum) temporal trend.
4. The overlap of these species groups at intermediate periods of time explains the peak of diversity after the disturbance. Thus, the diversity of species groups is an important driver of species richness.

## 2 STUDY SITE

**FIGURE 1:**
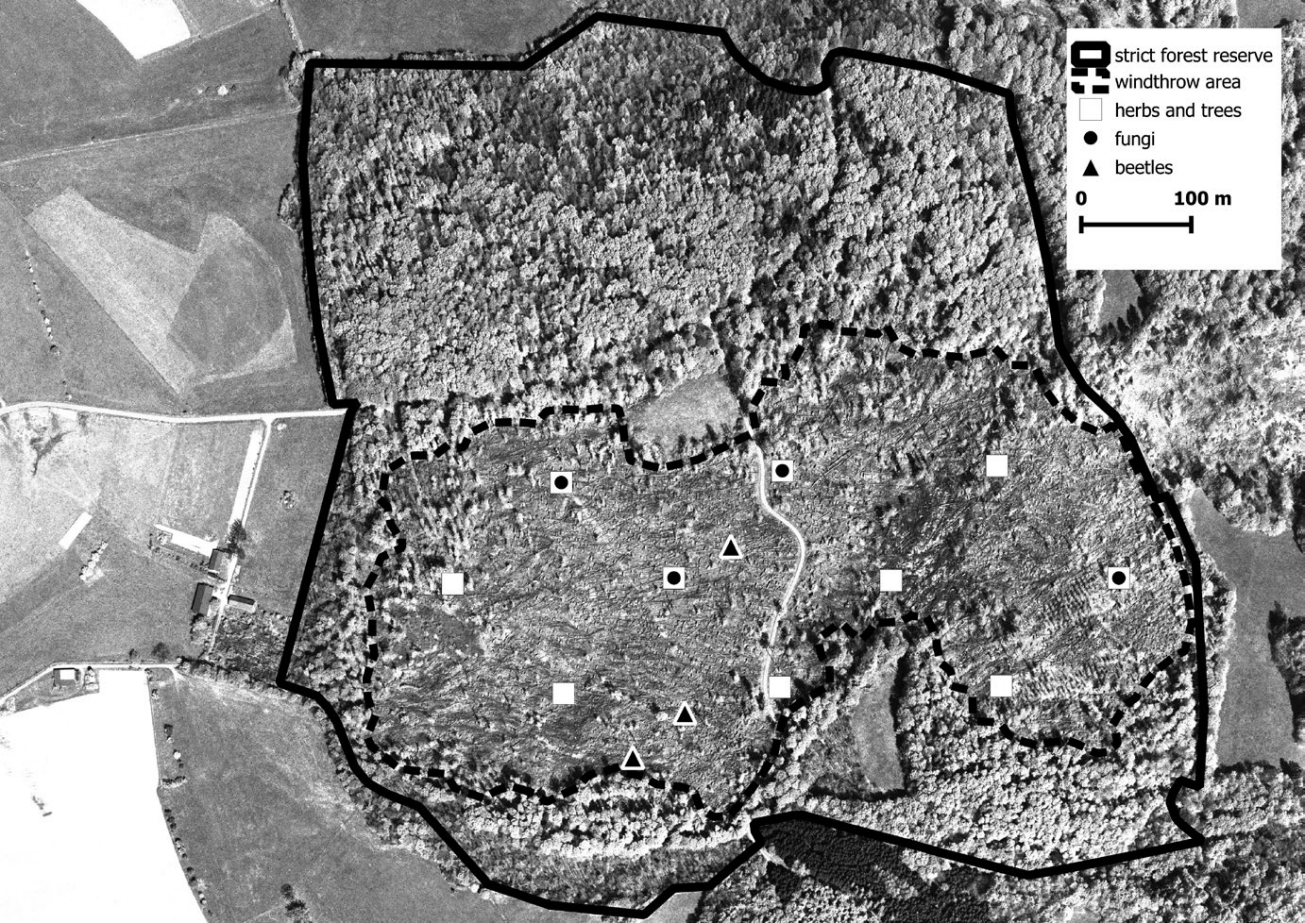
Map of the windthrow area and location of the study plots

The “Weiherskopf” reserve is situated in South Hesse, Germany. It consists of a 53 ha unmanaged strict forest reserve, of which 22 ha are windthrow areas resulting from the storms of February 1990 (Figure 1). The strict forest reserve lies between 310 m and 410 m above sea level in an area of basalt bedrock overlain with loess. The site is eutrophic with a balanced water regime. Annual precipitation is 970 mm, of which 390 mm falls in the vegetation period between May and September, while the mean annual temperature is 7.7°C, with a mean of 14.2°C in the vegetation period (1961–1991 reference period, Gauer & Aldinger, 2005). At the time of the windthrow event, the stand was a closed beech (*Fagus sylvatica*) forest with a 10% admixture of oak (*Quercus robur, Quercus petraea*), spruce (*Picea abies*), and a smaller proportion of other broadleaved species, in particular ash (*Fraxinus excelsior*), sycamore (*Acer pseudoplatanus*), and Norway maple (*Acer platanoides*). The beech stand was between 80 and 110 years old with a ground vegetation layer made up of *Fagion sylvaticae* alliance (cf. Mucina et al., 2016). The strict forest reserve “Weiherskopf” was set aside from forestry interventions in 1989. In 1987/88, 35 m^3^ ha^−1^ of wood was harvested in the area.

## 3 FIELD SURVEYS

Research on the effects of windthrow was conducted in the strict forest reserve “Weiherskopf” in two distinct phases. The first study phase, conducted under the auspices of the Hesse Forest Management Institute, Giessen, ran from 1990 to 2000, while in 2013/2014 a second survey phase was carried out by the North West German Forest Research Institute (NW‐FVA). All surveys relevant to this study were conducted in the 22 ha windthrow area of the strict forest reserve. The boundary of the windthrow area, mapped during the original survey in 1990, was adjusted slightly after comparing orthophotos from 1990 and 1993 and digital terrain models based on LIDAR laser scans from 2013, on which the root‐plate mounds from 1990 could still clearly be seen (Figure 1).

In the first phase, 10 permanent, square 400 m^2^ plots were established in the windthrow area, arranged with the upper left corner of the plots at every second node of a 100 m × 100 m monitoring grid. These plots were each divided into four 10 × 10 m quadrants, and surveys of tree regeneration and ground vegetation were conducted annually between 1990 and 1995 and then again in 1999 and 2007 (tree regeneration and tree layers only) in the northwest quadrant of each plot (Anonymus, 2007; Feldmann et al., 2009; Willig, 2002). Tree regeneration, per species and height class, was counted and recorded. All species in the ground vegetation (also including woody plants up to 50 cm high) were recorded with an estimated ground cover. While in the first survey tree regeneration was counted in 25 regularly spaced 1 m^2^ squares, from 1995, as the tree regeneration grew and thinned, the tree regeneration survey was expanded to include the entire 100 m^2^ northwest quadrant in each plot. The surveys in 2013 (forest structure and regeneration) and 2014 (ground vegetation) were conducted using the standard monitoring protocol for forest reserves in Hesse (Meyer et al., 2021). Circular 500 m^2^ plots, centered on the grid nodes, were used to survey forest structure (all standing trees (alive or dead) ≥7 cm diameter at breast height, lying trees ≥20 cm diameter at butt end). The ground vegetation as well as tree regeneration was surveyed in the 10 × 10 m plots of the first phase inventory. In order to include all tree layers, from regeneration to fully grown trees, in the data analysis also in‐grown trees (≥7 cm diameter at breast height) were added to the dataset. Results for forest structure and tree regeneration are standardized to per hectare quantities (number, volume).

Between 1991 and 2005, mycological surveys were carried out annually in 4 of the permanent 400 m^2^ plots. Each plot was visited at least once every month between May and October and all wood‐rotting fungi species (fruit bodies) occurring on standing but broken trunks, stumps, fallen trunks, branches, and woody debris of beech were recorded. All trunks within the plots were surveyed. In addition, in late summer, at least 16 trunk cross‐sections were taken from fallen beech trunks and analyzed in the laboratory (humid chamber incubation) for fungal infection and fungal species association (Willig & Schlechte, 1995). A further survey was carried out in 2008 (Keitel & Schlechte, 2009). For reasons of comparability between the plots, only two substrate classes were considered—lying beech logs (>20 cm diameter) and lying beech litter (5 mm < diameter < 20 cm), as only these two substrate types were recorded consistently in all surveys.

In order to sample saproxylic beetles, three trunk eclectors were placed on 1 m sections of lying, dead beech trunks between 1991 and 2000. The traps remained on the same trunk sections for the entire period (permanent plots). At the end of each survey year, they were moved temporarily to an adjacent trunk section, in order to allow for new colonization of the sample patch, before being repositioned on the original area for the next year. The trap contents were collected monthly during the summer period, but only one collection was made in winter each year (between November and March). The method is described in detail in Dorow (2002) and Flechtner (2002).

## 4 DATA ANALYSIS

Only those records were included in the data analysis which were determined up to the species level. In case of trees, we treated *Quercus petraea* and *Quercus robur* as one species. In case of birch, we assumed that all individuals were *Betula pendula*. In relation to arthropods, only beetles were considered.

All statistical analysis and graphical representations were produced using the statistical analysis system SAS 9.4 (SAS Institute).

Based on presence/absence data, mean alpha, beta, and gamma species richness (syn. diversity) were calculated per survey year for each of the four species groups—beetles, wood‐dwelling fungi, herbs, and woody plants (trees and tall shrubs) separately. Alpha diversity was defined as the number of species present per plot in a given year. Beta diversity was calculated for all pairwise plot combinations as the number of species unique to plot a or plot b in a given year (proc distance under SAS 9.4, method = overlap). Gamma diversity was defined as the total number of species present in a given year based on all plots surveyed.

Plotwise mean similarity of species composition with the first year of observation was calculated using the Jaccard index (proc distance under SAS 9.4, method = simratio).

In order to validate whether the change of species composition exhibits distinct successive phases, we applied a cluster analysis with Ward's minimum‐variance method (proc cluster under SAS 9.4, method = ward) and illustrated results in a clustering tree (proc tree under SAS 9.4).

The course of frequencies in the observation period was deployed to assign species to so called successional model types. Successional model types were defined as distinct temporal trends of frequencies after the disturbance event. We distinguished four model types, that is, increasing, decreasing, optimum (hump‐backed), and pessimum (concave). Charts of the course of frequencies over time were inspected by the authors P. Meyer, M. Schmidt, E. Feldmann, and R. Larkin independent from each other to assign one of the four types or, in case this was not considered adequate, the type “no model”. A final type was assigned if more than half (3 or 4) of the decisions matched. This was the case for 447 of 508 species evaluated. If less than 50% matched, the species was assigned the type “no model”. One of the four successional model types could be assigned to 273 of all 508 species determined (Table 2).

To test whether the overlap of successional model types is a significant driver of species richness, the Shannon index (Shannon and Weaver 1976) of successional model types (*H*′) was calculated for each species group based on the proportions of the species belonging to the respective type. Alpha diversity and gamma diversity were modeled as a function of *H*′ with a generalized linear mixed model (proc glmmixed under SAS 9.4). To account for temporal and spatial autocorrelation for alpha diversity, we employed plot number and year as random effects. For gamma diversity, the year of observation was implemented as random effect.

## 5 RESULTS

### 5.1 Regeneration and density of the tree layer

**FIGURE 2:**
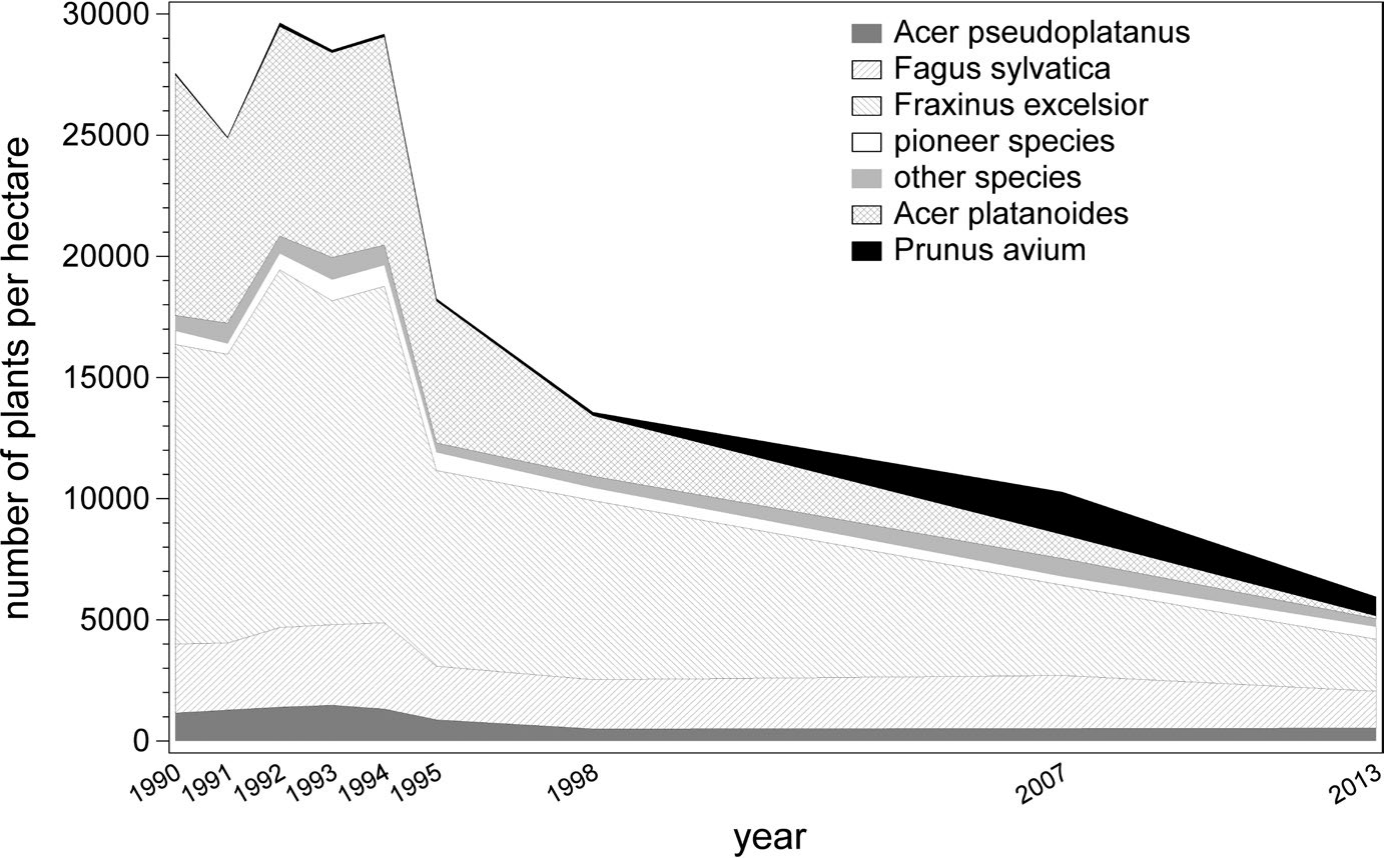
Development of tree number of regeneration (saplings and small trees and shrubs <7 cm dbh) per species or species group, respectively

Regeneration of the tree layer started immediately in the first vegetation period after the windthrow. This was possible because advance tree regeneration was present below the canopy at a high density of more than 27,000 plants per hectare (Figure 2). Even though beech had made up approx. 80% of the canopy before windthrow, it represented only 12% of the regeneration layer, which was dominated by ash (48%) and Norway maple (30%), with smaller amounts of sycamore maple (5%) and other species. Almost all of the regeneration was under 1.3 m high. By 1992, this figure had risen to around 29,000 trees per hectare and remained high until 1994 before falling sharply by 1995. In 2013, there were ca. 6,000 trees per hectare <7 cm diameter at breast height (dbh). There was a dramatic decrease in the proportion of ash and Norway maple during the observation period. In particular, the relatively fast‐growing ash passes the 7 cm dbh threshold earlier than the other species, thus outgrowing the regeneration layer. As a consequence, the stand ≥7 cm dbh was dominated by ash in respect of stem number (Table 1). The proportion of beech increased steadily from 1995, and in 2013 made up ca. 40% of stems >3 m height and <7cm dbh.

**TABLE 1:** Mean values and standard deviation of density parameters of the stand ≥7 cm dbh in the windthrow area in 2013

Species	Living stand ≥7 cm dbh		Deadwood
	*n* ha^−1^	m^3^ ha^−1^	*n* ha^−1^
*Fraxinus excelsior*	835 ± 841	67 ± 64	31 ± 48
*Fagus sylvatica*	196 ± 263	76 ± 93	2 ± 6
*Acer pseudoplatanus*	153 ± 231	18 ± 33	0 ± 0
*Acer platanoides*	95 ± 120	8 ± 12	0 ± 0
Other tree species	73 ± 176	6 ± 12	9 ± 30
*Salix caprea*	64 ± 107	10 ± 15	4 ± 8
*Prunus avium*	60 ± 66	11 ± 10	2 ± 6
*Picea abies*	24 ± 49	2 ± 4	2 ± 6
*Carpinus betulus*	16 ± 20	2 ± 5	0 ± 0
*Populus* × *canadensis*	7 ± 24	4 ± 12	0 ± 0
*Quercus robur*/*petraea*	2 ± 6	0 ± 0	0 ± 0
Sum	1,524 ± 743	203 ± 84	49 ± 61

Note

Results of 10 circular sample plots spaced systematically in a 100 × 100 m grid. Living stand: *n* ha^−1^ and m^3^ ha^−1^ = stem number and volume per hectare, deadwood: *n* ha^−1^ = number of snags and standing dead trees per hectare.

Although there is no information for standing wood volume in the windthrow area immediately before the disturbance, figures from a survey in the unaffected part of the strict forest reserve from 1991, one year after the windthrow event, give an approximate picture of the original stand characteristics. There, the volume of live wood with dbh ≥7 cm was 327 m^3^/ha, with 676 trunks ha^−1^. Beech made up ca. 75% of the stems, with hornbeam (8%), spruce and other conifers (6%), sycamore (4%), and ash (2%). Beech accounted for 63% of the wood volume, followed by spruce (6.7%), sycamore (5.5%), oak (4.5%), hornbeam (3.4%), and ash (3%). Other conifers accounted for ca. 11% of the wood volume.

By 2013, the stand characteristics in the recovering windthrow area were very different. The volume of live wood with dbh ≥7 cm in the windthrow area was already 217 m^3^ ha^−1^, with 1,524 trunks ha^−1^ on average (Table 1). Ash made up the bulk of the stems (54%), followed by beech (13%) and sycamore (10%). In terms of wood volume, beech and ash were similarly dominant because some mature beech had remained standing after the windthrow event. Total mean cover of the tree layer was, in the 2014 vegetation survey, estimated to be 85%. In 2014, there were only 7 tree species in the overstory, with no more than four in a single plot. Wild cherry (*Prunus avium*), which made up an increasing share of the regeneration from 1998 until 2007, is present in the subcanopy in 4 plots and shares dominance with beech in one. In the 4 plots which are dominated by ash, there is no beech with a diameter at breast height over 7 cm. Beech is strongly dominant in 3 plots and codominant in 3 others.

There were, on average, 49 snags per hectare, with an estimated volume of 3 m^3^ ha^−1^ (Table 1) and a mean of 218 lying deadwood trunks per hectare (results not shown), with a volume of 110 m^3^ ha^−1^, all of which were in an advanced state of decay in 2013.

### 5.2 Development of species richness

**FIGURE 3:**
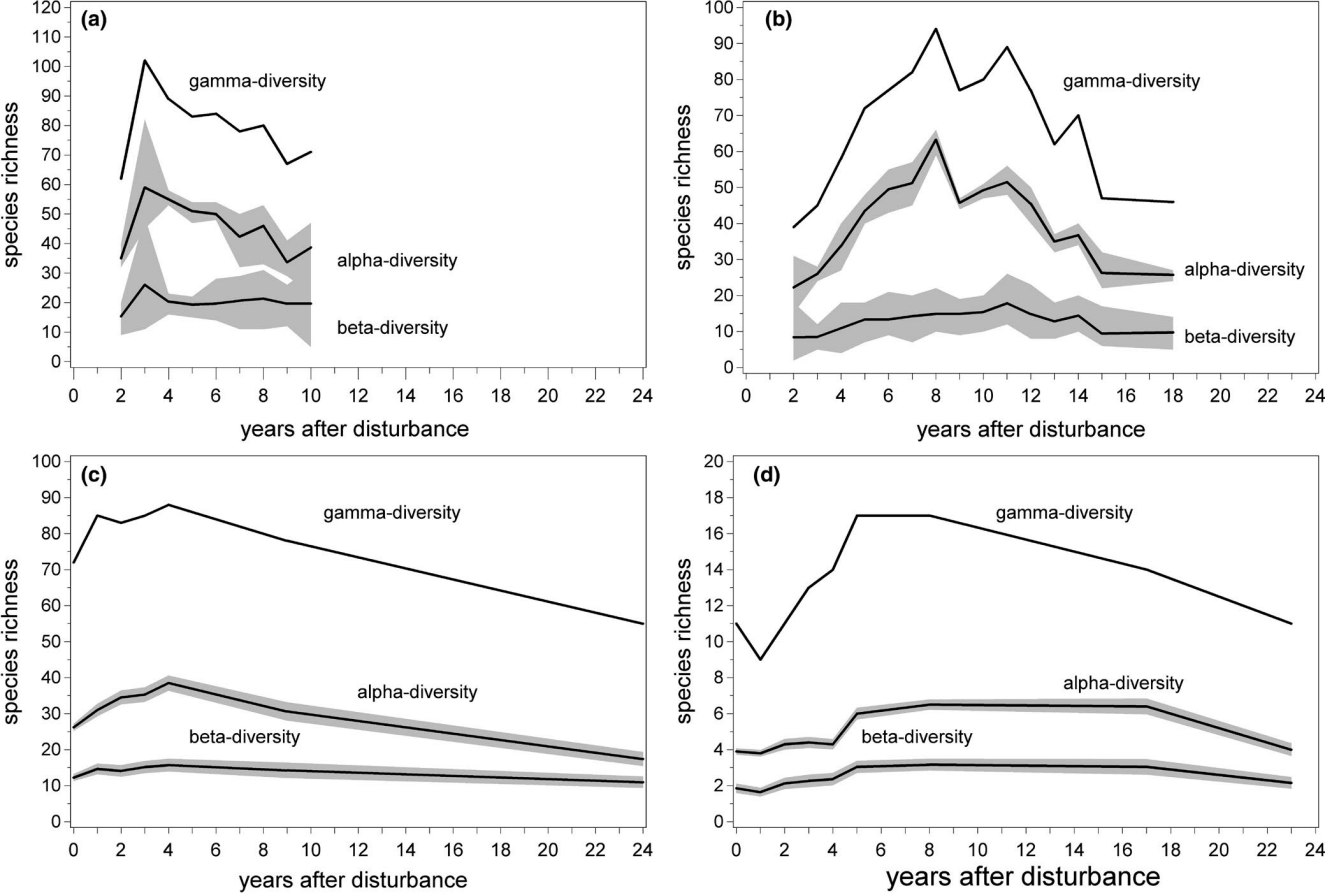
Development of species richness (alpha, beta, and gamma diversity s. text) with time elapsed since 1990 for (a) beetles, (b) fungi, (c) herbs, and (d) trees. The line indicates the mean of plots (alpha), pairs of plots (beta), or overall (gamma) species richness. Shaded area: 95% confidence interval (herbs and trees) or range (beetles, fungi)

Over the period studied, 508 different species were recorded, with 219 species of beetles, 150 wood‐dwelling fungi, 121 herbs, and 18 trees and shrubs (Table 2). In all species groups, γ‐diversity and α‐diversity followed a hump‐backed or optimum curve (Figure 3). Species richness of beetles peaked 3 years after the windthrow followed by the peaks of herbs and trees after 4 and 5 years, respectively. Fungal richness peaked later, at 8 years after the disturbance. Only the curve for wood‐dwelling fungi was more or less symmetric. In all other species groups, a considerable left skewed curve with a long tail of slower decrease was observable. In the case of beetles and fungi, diversity was higher at the end of the observation period than at the beginning. Tree species richness fell back to the initial level of 11 species in all ten plots at the end of the observation period. In the case of herbs, richness fell markedly below the initial value by the end of the observation period. In contrast to α‐ and γ‐diversity, β‐diversity exhibited a less pronounced optimum or a nearly flat temporal course. Thus, overall γ‐diversity was mainly driven by plotwise richness, rather than by between plot differences in species assemblages.

**TABLE 2:** Number of species per group and model type. Model type refers to the temporal frequency trend after the disturbance event

Model type	Beetles	Fungi	Herbs	Trees	All
Increasing	15	13	3	1	32
Decreasing	13	11	17	1	42
Optimum	58	81	50	9	198
Pessimum	1	0	0	0	1
No model	132	45	51	7	235
Sum	219	150	121	18	508

### 5.3 Temporal course of similarity

**FIGURE 4:**
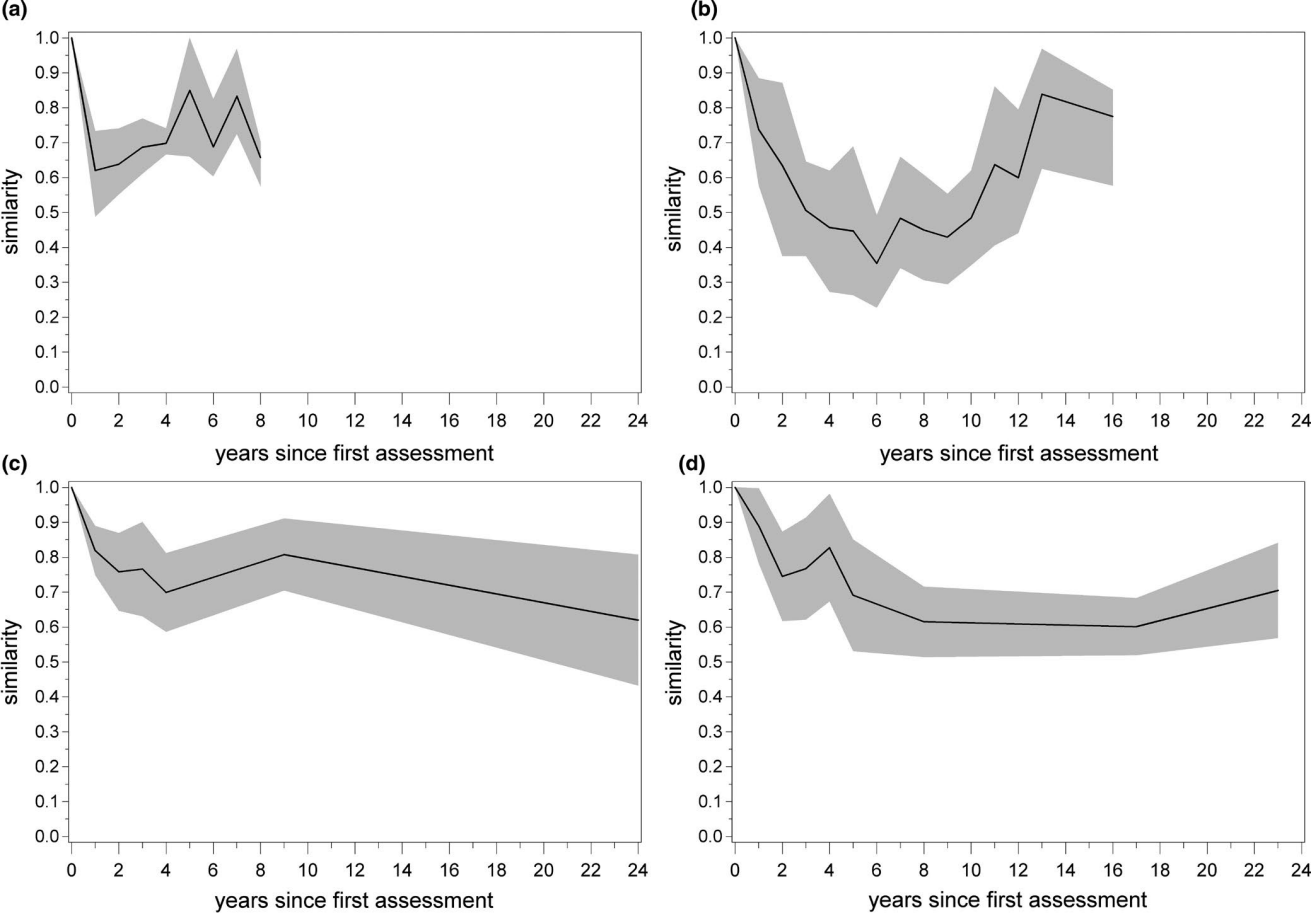
Similarity (Jaccard index) of species composition to the initial year of assessment for (a) beetles, (b) fungi, (c) herbs, and (d) trees. Shaded area: 95% confidence interval

While the temporal course of species richness conformed to the hump‐backed curve expected under the IDH, the similarity of species composition did not confirm the hypothesis. Instead of exhibiting a continuous decrease, in all groups similarity converged to the initial state after a first decreasing phase (Figure 4). The groups differed in respect of the subsequent course of similarity. The beetle community showed high interannual variation but no clear trend in this third phase of development. The fungal community returned continuously to the initial composition, showing only two development phases of similarity. Species composition of herbs showed a third phase of decreasing similarity, while with respect to trees there were two phases of increasing similarity separated by a phase where species composition did not change. Overall, there was no indication of a clear successional trend of species composition. Instead, diverse courses of development were found, decreasing, increasing, and constant phases with no detectable change.

### 5.4 Clusters of years

**FIGURE 5:**
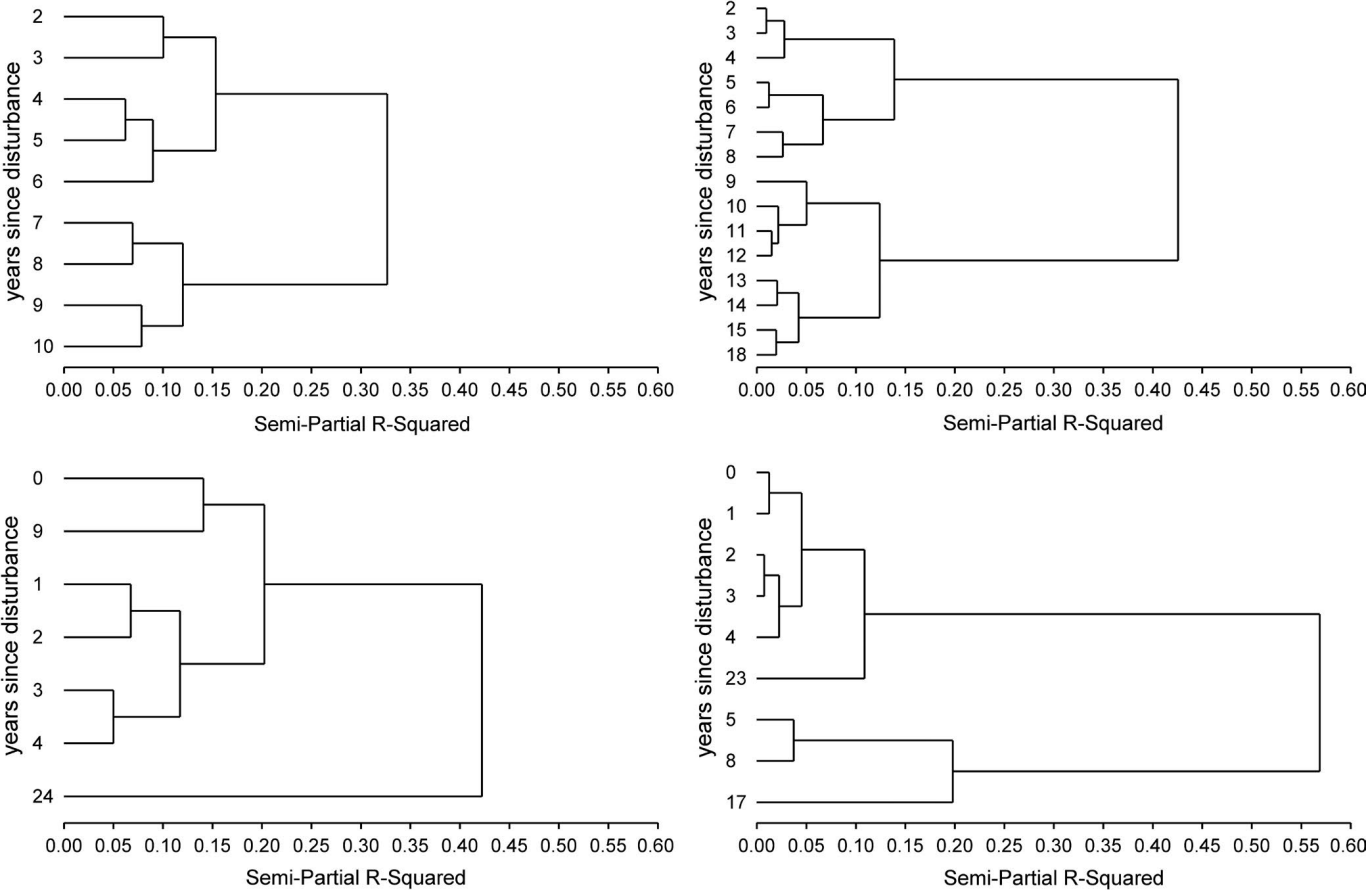
Clusters of the species composition to years after disturbance for beetles (upper left), fungi (upper right), herbs (lower left), and trees (lower right)

The development patterns of similarity suggested different phases of species composition. Phases are understood as groups of years which can be separated from earlier and later years equally. The cluster analysis revealed that these groups can in fact be determined, though they do not in all cases follow the temporal sequence of years (Figure 5). In the beetle community, the years 2 to 6 after the windthrow were clearly separated from the years 7 to 10. At first sight, it was surprising that, although similarity to the initial composition increased markedly in the fungal community 6 years after the first assessment (equals 8 years after the windthrow), there was a distinct divide in the chronologically ordered clusters of survey years at this point. This can be explained by the fact that the species composition in the first phase of decreasing similarity differs from the second phase of increasing similarity. Different species combinations can result in equal similarity values in relation to the initial community. In the case of herbs and trees, clusters did not consequently follow a chronological sequence. For herbs, the year of disturbance and the ninth year after disturbance constitute one cluster. This indicates that the species composition converged to the initial state after 9 years, a result also reflected in the course of similarity (Figure 4). 24 years after the windthrow, the species assemblage had, however, receded markedly from the initial composition. This trend is also reflected in the distinct decrease in similarity between years 9 and 24 after disturbance (Figure 4). The tree community paralleled the clustering pattern of the herbal community in that it exhibited a converging phase. An important difference is that tree species composition converged at the end of the observation period to the initial state and not in between. The maximum distance to the initial community was reached 17 years after the windthrow.

### 5.5 Successional model types

The determination of successional model types was successful for more than half of all species (Table 2). The proportion of the group “no model” was highest in beetles (60%), followed by herbs (42%). For fungi, 70% of species could be assigned to a successional model type, while for trees the proportion was nearly 61%.

Shannon diversity of the successional model types proved to be a significant predictor for species richness at the plot (alpha) level for all species groups except for beetles (Table 3). In contrast, there was not a significant effect of the diversity of successional model types on gamma diversity, except for a minor effect in case of fungi (Table 4).

**TABLE 3:** Results of generalized linear mixed models for alpha diversity of a species group as a function of the Shannon diversity (*H*′) of the successional model types within this group

Model type	Intercept		*H*′ successional model types	
	Estimate	*p* > *t*	Estimate	*p* > *t*
Beetles	137.6	.0155	10.6	.6230
Fungi	−19.5	.4066	82.1	.0001
Herbs	24.5	.0009	10.5	.0326
Trees	3.2	.1369	12.3	<.0001

Note

Given are estimates for the intercepts, the coefficient of the alpha diversity, and the values for *p* > *t*.

**TABLE 4:** Results of generalized linear mixed models for gamma diversity of a species group as a function of the Shannon diversity (*H*′) of the successional model types within this group

Model type	Intercept		*H*′ successional model types	
	Estimate	*p* > *t*	Estimate	*p* > *t*
Beetles	83.7	<.0001	−6.1	.58441
Fungi	−79.0	.17967	139.0	.02106
Herbs	87.0	.19594	−2.6	.96381
Trees	12.1	.00842	1.2	.76169

Note

Given are estimates for the intercepts, the coefficient of the Shannon diversity of the successional model types, and the values for *p* > *t*.

## 6 DISCUSSION

Our study found that temporal change in species richness followed the hump‐backed pattern predicted by the IDH, but also revealed different relationships for the four featured species groups in respect of the similarity of community composition over time and the role of successional model types in explaining species richness. There were also differences in the timing of peak richness and in the rate of decline of species richness between the groups.

The change of species richness and composition after disturbances is determined, in case of plants, by the composition of the soil seed bank and in general by the rates of immigration of new species and the local extinction of surviving species (Collins et al., 1995). The factors that drive these increasing and decreasing processes can be assumed to depend strongly on the traits of the species involved, especially in respect of resource requirements, competitive strength and the ability to colonize a new site and to coexist with or even facilitate other species. Furthermore, it has been shown that intermediate‐severity disturbances increase the substrate heterogeneity (Hart & Kleinman, 2018), thereby creating a high diversity of ecological niches suitable for colonization by new species. Not all colonizing species arrive in a newly disturbed site at once but, with time, more species have a chance to establish. Thus, the time taken for a saturation of the species pool must also be considered, in order to understand the development of species richness and composition after disturbance.

### 6.1 Confirmed and rejected hypothesis

Although the hump‐backed pattern was prominent for alpha and gamma diversity, it was barely detectable for beta diversity, indicating that, in the studied stand, the change in species composition was highly synchronized over different plots or logs, respectively. The compact shape of the small windthrow area of 22 ha and a rather homogenous biotope structure (formerly managed beech forest) may have facilitated commonality in our study. Plot fungal species composition, for instance, seems to be similar only where there were short distances between plots (Abrego & Salcedo, 2014), while, where saproxylic beetle assemblages differ significantly, the studied plots are regionally scattered (Floren et al., 2015; Purahong, Wubet, Krüger, et al., 2018). Constant beta diversity may also be due to a species pool that has been reduced by management activities or landscape fragmentation. Halme et al. (2013) found local fungal beta diversity to be low in previously managed forests during the entire decay process, whereas in natural forests it increased with advancing deadwood decay.

Where the IDH pattern is evident, it was expected that a linear succession would occur, driven by increasing competition with time and indicated in our analysis by steadily decreasing similarities. However, after an initial decreasing phase, a partial recovery of the initial species composition was observed in all species groups.

For fungi, herbs, and trees, the diversity of successional model types increased alpha species richness. However, the effect on gamma diversity was either not significant or weak. We had to reject the respective hypothesis at the plot (alpha) level for beetles and for all species groups except for fungi at the level of overall (gamma) diversity. Therefore, despite the hump‐backed course of species richness over time, it was not possible to derive an explanatory model that applies to all species groups equally.

### 6.2 Specific considerations per species group

In order to understand our results, it was necessary to look at the ecological differences between the species groups and how ecological conditions change after the disturbance.

The immediate effect of a windthrow is the sudden creation of abundant fresh substrate available for colonization by a range of organisms. At the same time, the site is opened, facilitating the influx of propagules from outside the area and allowing in light. Despite the potential for colonization by pioneers, for tree species this process was of secondary importance compared with the growth of advanced regeneration. The development of the tree layer strongly influences the ecological conditions for the other species groups. Because the regeneration layer remained relatively intact, this legacy of the previous stand largely determined development after the disturbance.

The species richness and composition of the herbal community are assumed to be mainly influenced by light conditions, water and nutrient supply, processes activating diaspores in the seedbank, and the amount of exposed mineral soil suitable for colonization (Oheimb et al., 2007). With time, tree regeneration limits the availability of light, water, and nutrients for the herb layer and the growing tree regeneration cohort itself, resulting in high rates of self‐thinning (Zeide, 2005), which are indicated by the steep decrease in tree density over time. It further provides higher air humidity and a more balanced temperature regime by shading (Abd Latif and Blackburn, 2009; de Freitas & Enright, 1995; Forrester et al., 2012), while the area of uncolonized exposed mineral soil also diminishes. Therefore, with ongoing tree succession the heterogeneity and amount of key resources are decreasing strongly. Species richness of herbs and trees peaked around five years after the disturbance. From this point on, it can be assumed that, by limiting resource availability, the dense tree layer caused species losses in both the herb layer and within the tree cohort itself.

The disturbance caused a major shift in tree layer composition from a beech‐dominated mature forest to an ash‐dominated pole phase stand. While the tree community started to return to the initial species composition, the herbal community moved further away from the previous state. It is assumed that anthropogenic disturbances, linked to management activities before the site was designated a strict forest reserve, had slightly increased herb species numbers. In 1987/88, 35 m^3^ wood per hectare had been harvested on the site, which would have opened up the tree canopy. The dense tree regeneration cohort, growing up after the windthrow, then resulted in less light availability on forest floor than immediately prior to the windthrow.

Early saproxylic fungal and beetle colonizers are highly specialized (Wende et al., 2017), but weak competitors are rapidly replaced by secondary colonizers as suitable substrate for specialists is consumed or decays (Boddy, 2001; Purahong, Wubet, Lentendu, et al., 2018; Ulyshen & Hanula, 2010; Wermelinger et al., 1999). Beetle life cycles are, however, adapted to temporary resource availability. Bark beetle adults emerge in spring in great numbers to emigrate in search of new host trees. The sharp increase in beetle species richness in the first 3 years followed by a decline to year 5 observed in this study is a pattern which has also been shown in other studies (Bouget & Duelli, 2004; Wermelinger et al., 2002; Weslian et al., 2011). Because many species of fungi are specialized in differently decayed wood (Boddy, 2001; Ulyshen & Hanula, 2010), the community of saproxylic fungi undergoes a self‐induced compositional change with progressive wood decomposition. Saproxylic beetles go through similar, dependent, compositional changes, with bark beetles gradually being replaced by wood boring beetle species. As log decay continues and the wood is more completely colonized by fungi, fungivores beetles dominate in the later decay stages. These phases are gradually overlapping rather than discrete (Wermelinger et al., 2002).

Fungal species richness peaked 8 years after disturbance. At this stage, most deadwood >10 cm diameter would have been in intermediate decay stages and the pieces <10 cm in advanced decay, with the bark on smaller pieces almost completely decayed (Herrmann et al., 2015; Müller‐Using & Bartsch, 2009). In the intermediate decay stage, the greatest variety of patches in different decay stages exists simultaneously. The heterogeneity in this stage is known to support the greatest fungal species richness (Heilmann‐Clausen & Christensen, 2003; Jönsson et al., 2008).

The results of this study, that fungal species richness increases with increasing diversity of successional model types, implying that species characteristic of different phases are simultaneously present, fit well with these observations.

As decay progresses, deadwood becomes increasingly homogenous, supporting a diminishing number of niches, especially when logs collapse and lie completely on the ground. This homogenization has been shown to be the main cause of decreasing richness of fungal species with progressing wood decay (Jönsson et al., 2008). A lack of bark cover and the low number of resident fungal species also greatly reduces habitat and resource availability for many beetle species (Brunet & Isacsson, 2009).

The rapidly developing tree layer can also profoundly affect the species assembly of saproxylic organisms. Beetle and fungal richness are greater in exposed deadwood (Brunet & Isacsson, 2009; Seibold et al., 2016; Toljander, 2006). Fungal and beetle species communities in gaps and under canopy have been found to differ significantly (Brazee et al., 2014; Odor et al., 2006; Seibold et al., 2016). Thus, canopy closure will negatively affect the initial community, while constant temperatures lead to dominance by competitive fungal species (Toljander, 2006).

The rapid development of the tree layer in this study is, however, a legacy of previous forest management. Where advanced regeneration is lacking or site conditions favor herb and grass species, tree regeneration may happen much more slowly (Klopčič et al., 2015; Kompa und Schmidt, 2005; Topoliantz & Ponge, 2000).

### 6.3 Community dynamic

Late succession forest herbs and tree species do not tend to form persistent seed banks (Tiebel et al., 2018; Wódkiewicz & Kwiatkowska‐Falińska, 2010), while ruderal and pioneer herb species do (Doelle & Schmidt, 2009; Halpern et al., 1999). Although primary fungal colonizers can already be present as latent propagules in living trees, fungal afflictions tend to be rare in managed forest (Bässler et al., 2012; Heilmann‐Clausen & Christensen, 2003). Most fungi probably arrived as spores very soon after the trees were downed (Boddy, 2000) or were carried by saproxylic beetle vectors. Saproxylic beetles actively seek out coarse woody debris, attracted by volatile chemicals released from the freshly dead wood (Bouget & Duelli, 2004).

Activation of latent fungal propagules and the soil seed bank, as well as colonization from outside, all probably contributed to the increase in species on the rising arm of the hump‐backed curve. During this phase, similarities decreased in our analysis, reflecting both the gradual elimination of less competitive pioneer species and the immigration of mobile species. As cover increases, immigration becomes less important, as even highly competitive species can find it hard to establish on occupied substrate (Boddy, 2000; Cordonnier et al., 2006). Inter‐ and intraspecific competition among resident species will then become more important. Competition is especially fierce in the tree regeneration cohort after canopy closure and can be assumed to be a major driver for shifts in tree species composition (Klopčič et al., 2015). Competition is mitigated, however by substrate and resource heterogeneity, which, in an unsalvaged windthrow, provide a large variety of available niches. Facilitation also becomes more important as the number of resident species increases and has been noted in all the studied species groups, usually as a response to stress factors (Heilmann‐Clausen & Boddy, 2005; Weslian et al., 2011). As such, facilitation is probably most effective in promoting species coexistence as immigration slows. As the level of stress becomes more severe, facilitation gives way to competition. Species with a conservative resource‐use strategy, however, may still be facilitated by neighbors, even under conditions of high stress (Fichtner et al., 2017).

As available resources become scarce and substrate more homogeneous, competitive exclusion is expected to reduce the number of species present. However, even in late successional communities, microclimatic and microtopographic niches mean that species with different strategies can coexist (Halpern, 1989; Heilmann‐Clausen, 2001). Environmental modification and selective competitive pressure, coupled with different dispersal speeds, mean that colonization can still occur (Halme et al., 2013; Li et al., 2015).

### 6.4 Methodological limitations

Although our study is somewhat unique in respect of its duration and the fact that different species groups are covered, there are some methodological limitations needing consideration. In particular, the number of plots/traps in case of fungi and beetles was rather low. Furthermore, it was disadvantageous that no surveys were conducted for fungi and beetles in the first two years after windthrow. These shortcomings underline the well‐known challenges of long‐term observational studies, especially when species‐rich groups are observed (Lindenmayer & Likens, 2010; Meyer, 2020). We conducted an observational study and had to rely on heuristic reasoning to reveal the importance of ecological factors. With an experimental approach, different factors could be separated on a sound statistical basis. However, experiments suffer from the general limitation that, within a reasonable frame of time and money, they can hardly reflect the complexity of real‐world settings. This limits the transferability of experimental results to a complex reality (Hilborn & Mangel, 1997) and lends support to the value of long‐term observational studies.

## 7 CONCLUSIONS

Organisms have evolved different life strategies which enable them to establish and survive in particular environments and species assemblages (Grime, 1977; MacArthur & Wilson, 1967). In the original derivation of the IDH, Connell (1978) assumed that competitive exclusion eventually leads to dominance by superior competitors, that is, that one strategy will increase at the expense of all others.

Although resource competition is a factor in species assemblage change in all species groups (Boddy, 2000; Grime, 1977; Leuschner, 2015; Newman, 1973; Petritan et al., 2007; Schlyter & Anderbrant, 1993), the results of this study indicate that other factors also have to be taken into consideration, even if the hump‐backed course of the diversity–disturbance relationship over time suggests the traditional IDH explanation. The IDH has been described as an umbrella concept, encompassing patterns generated by disparate phenomena (Collins & Glenn, 1997; Miller et al., 2011). Our results underline that the mechanisms driving the increase and decline in species richness after disturbances are complex vary with species group and that many different factors are involved: the disturbance itself, legacy effects, colonization, competition and facilitation, habitat heterogeneity, and random saturation processes of the species pool.

We found indications for a strong effect of competition in case of trees and herbs. For fungi and beetles, substrate heterogeneity and microclimate seemed to be more important. Thus, we conclude that disturbances contribute to increasing species richness not only by reducing the effectiveness of competitors but also by increasing the amount and diversity of resources, as well as their rate of change over time.

Changing disturbance regime is likely to be among the most profound effects that climate change will have on forests in the next decades (Seidl et al., 2017; Senf & Seidl, 2020). Improved understanding of the processes and patterns in species response to disturbance in forest ecosystems is becoming increasingly important, especially with a view to implementing disturbances consciously in management concepts (Bollmann & Braunisch, 2013; Meyer & Ammer, 2019).

Our study demonstrated the importance of untreated windthrow areas for restoring and conserving species diversity also for European beech forest. The large amount of deadwood, structural complexity, microtopographic, and microclimatic variety provide opportunity for many species to establish. Removing all windthrown trees, clearing the ground and / or replanting would considerably reduce site heterogeneity and available habitat or the time available for succession.

## CONFLICT OF INTEREST

The authors confirm that they do not have any conflicts of interests to declare.

## AUTHOR CONTRIBUTIONS

**Peter Meyer:** Conceptualization (lead); Data curation (lead); Methodology (lead); Visualization (lead); Writing‐original draft (equal); Writing‐review & editing (supporting). **Marcus Schmidt:** Conceptualization (supporting); Formal analysis (equal); Methodology (supporting); Visualization (supporting); Writing‐review & editing (supporting). **Eike Feldmann:** Conceptualization (supporting); Investigation (equal); Methodology (supporting); Validation (equal); Visualization (supporting); Writing‐original draft (supporting); Writing‐review & editing (equal). **Jürgen Willig:** Conceptualization (equal); Funding acquisition (lead); Investigation (equal); Methodology (equal); Project administration (lead); Writing‐original draft (supporting); Writing‐review & editing (equal). **Robert Larkin:** Conceptualization (supporting); Validation (equal); Writing‐original draft (equal); Writing‐review & editing (lead).

## Data Availability

We have archived the data in a DRYAD archive (https://doi.org/10.5061/dryad.x69p8czjr).
